# A hidden burden of neonatal illness? A cross-sectional study of all admissions aged less than one month across twelve Kenyan County hospitals

**DOI:** 10.12688/wellcomeopenres.13312.1

**Published:** 2017-12-18

**Authors:** Georgina A.V. Murphy, Vivian N. Nyakangi, David Gathara, Morris Ogero, Mike English, Clinical Information Network

**Affiliations:** 1 Centre for Tropical Medicine and Global Health, Nuffield Department of Medicine, University of Oxford, Oxford, UK; 2 Health Services Research, Kenya Medical Research Institute/Wellcome Trust Research Programme, Nairobi, Kenya

**Keywords:** Newborn health, neonatal care, paediatrics, newborn mortality, small and sick newborns, facility-based care, Kenya, Africa, low-resourced settings, global health

## Abstract

**Background:** Small and sick newborns need high quality specialised care within health facilities to address persistently high neonatal mortality in low-income settings, including Kenya.

**Methods: **We examined neonatal admissions in 12 public-sector County (formerly District) hospitals in Kenya between November 2014 and November 2016. Using data abstracted from newborn unit (NBU) admission registers and paediatric ward (PW) medical records, we explore the magnitude and distribution of admissions. In addition, interviews with senior staff were conducted to understand admission policies for newborns in these facilities.

**Results: **Of the total 80,666 paediatric admissions, 28,884 (35.8%) were aged ≤28 days old. 24,212 (83.8%) of newborns were admitted to organisationally distinct NBU and 4,672 (16.2%) to general PW, though the proportion admitted to NBUs varied substantially (range 59.9-99.0%) across hospitals, reflecting widely varying infrastructure and policies. Neonatal mortality was high in NBU (12%) and PW (11%), though varied widely across facilities, with documentation of outcomes poor for the NBU.

**Conclusion: **Improving quality of care on NBUs would affect almost a third of paediatric admissions in Kenya. However, comprehensive policies and strategies are needed to ensure sick newborns on general PWs also receive appropriate care.

## Introduction

Access to basic but high quality inpatient neonatal services for small and sick newborns will be key if progress is to be made in reducing neonatal mortality in low- and middle-income countries (LMICs) ^1, 2^. Care should ideally be in a newborn unit (NBU) with specialised equipment and staff providing interventions such as feeding and respiratory support and phototherapy ^2, 3^. However, evidence from single-site studies in Kenya and other resource-limited settings suggests that newborns are often admitted to general paediatric wards (PWs) due, among other reasons, to limited space and resources within the NBU ^4, 5^. Little is quantitatively known about this neonatal population, neither their magnitude nor characteristics, due to poor information systems ^6– 8^. This hidden population of neonatal patients may not benefit from specialised care or quality improvement efforts and may be missed in national statistics used to inform policy and planning.

We set out to explore the burden of neonatal admissions and the distribution of these admissions between NBUs and PWs across 12 County (formerly District) hospitals in Kenya. We further explored what might influence where care is provided for newborns in these hospitals. Our aim is to provide preliminary data to inform thinking on how best to organise comprehensive neonatal services in Kenya and potentially other LMICs.

**Box 1**

What is already known about this topic?

- Inpatient care in a specialised environment is important in efforts to reduce persistently high neonatal mortality in low-income settings.
- Single sites studies suggest that newborns can make up a high proportion of total paediatric admissions to hospitals in Kenya.
- Anecdotal evidence suggests newborns are sometimes admitted to general paediatric wards where they may receive less specialised care.

What does this add?

- Across 12 Kenyan County hospitals newborn admissions represented over a third of all paediatric admissions and 16.2% of newborn admissions were to paediatric wards.
- Little consistency was observed in admission policies across these large public sector hospitals, though most hospitals had a policy of admitting newborns from the community to the paediatric ward rather than the newborn unit. The physical infrastructure of a hospital may influence their admission policies.
- Clearer agreement on the best way to organise neonatal services is needed to ensure small and sick newborns receive care in the most appropriate setting with access to specialised services.

## Methods

This study was conducted across 12 County (formerly District) hospitals in Kenya, which form part of the Clinical Information Network (CIN) ^9, 10^. Data abstracted from medical records for all admissions to the PW between 1st November 2014 and 30th November 2016 were included in the study. Procedures for such data collection have been previously described ^9, 10^. Additionally, information about admissions to the NBU was retrospectively abstracted from admission registers at each hospital for the same time period by the same data clerks during February-June 2017. All data entry followed strict standard operating procedures and employed purpose-designed standardised data capture tools created in REDCap. Inbuilt range and validity checks and pre-designed cleaning scripts were run daily and weekly, respectively, on aggregate data with corrections made, where possible, by referring back to source documents. 

Information on admission policies for neonatal patients was obtained through telephone discussions with the nurse in charge and paediatrician at each health facility in April 2017 ( Supplementary File 1).

Data analysis was conducted in R statistical software version 3. Neonates were defined as patients aged ≤28 days old.

### Ethical statement

Scientific and ethical approval for the study was obtained from the Kenya Medical Research Institute National Scientific and Ethical Review Boards (SERU protocol number 3459), and study hospitals provided assent for inclusion of their data in the study.

## Results

**Figure 1.**
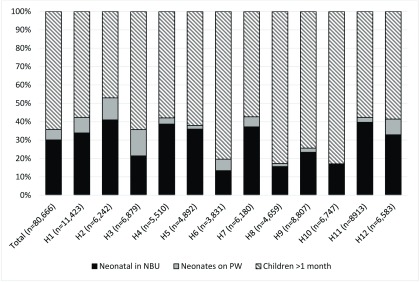
Neonatal and paediatric admissions to newborn units (NBU) and paediatric wards (PW) in County hospitals in Kenya, as a proportion of total paediatric admissions.

A total of 80,944 children and newborns were admitted, 56,732 to the PW and 24,212 to the NBU, between 1st November 2014 and 30th November 2016 in the 12 County hospitals. After exclusion of the 278 PW admissions with no recorded age, 80,666 (99.7%) admissions were included in this analysis. Figure 1 describes the distribution of these admissions for each hospital by patient group (neonate or older child) and ward (NBU or PW).

### Neonatal admissions

The total number of neonatal admissions to the 12 County hospitals was 28,884, representing 35.8% of all paediatric admissions; 83.8% (n=24,212) were admitted to the NBU and 16.2% (n=4,672) to the PW. The number of neonates admitted to the PW as a proportion of total neonatal admissions ranged from 1.0% (H10:11/1,152) to 40.1% (H3:987/2,459). Neonates accounted for 8.3% (4,672/56,454) of admissions to the paediatric ward. This proportion ranged from 0.2% (H10:11/5606) to 20.4% (H2:750/3,685).

Among neonatal patients admitted to PWs, mortality was 11.1% (519/4,672); 4.9% (229/4,672) were referred and the remaining 84.0% (3,924/4,672) were discharged home. Outcome was reported for only 69.0% (16,699) of patients admitted to NBUs. Of those with a recorded outcome, mortality was 12.3% (2,052/16,699), 2.1% (347/16,699) were referred, and 85.6% (14,289/16,699) were discharged home.

### Admission policies

Only nine of the 12 hospitals reported having a policy on admitting neonates to PWs ( Table 1). The three hospitals that reported only admitting neonates to their NBUs, reported having a cubicle in the NBU for isolation of sick neonates from the community (‘outborn’ ─either born at home or discharged home after birth). All other facilities reported admitting outborn newborns to the PW or in two cases (H4&H7) admitting this group to either the PW or NBU depending on their age and illness. Neonates born preterm (<36 weeks of gestational age) who required admission from the community were admitted to NBUs in most hospitals ( Table 1). Neonates who were referred from other hospitals were admitted to NBUs in 8/12 hospitals. In one hospital (H2) referred neonates who were diagnosed with sepsis were admitted to the PW and those without sepsis were admitted to NBU.

**Table 1.** Admission patterns of neonates across different hospitals.

	Physical size of NBU	Location of NBU	Neonates on PW	Inborn	Out-born/ Readmissions	Out-born & preterm	Referred
**H1**	Large	Designated	Separate room	**NBU**	**PW**	**PW/NBU ƚ**	**NBU**
**H2**	Large	Designated	All children <1 year in separate room	**NBU**	**PW**	**NBU**	**PW/NBU §**
**H3**	Small	In PW	Separate room	**NBU**	**PW**	**REFER**	**NBU**
**H4**	Large	Designated	Cubicle	**NBU**	PW/NBU *	PW/NBU *	PW/NBU *
**H5**	Medium	Designated with isolation room	Mixed with older children	**NBU**	**NBU**	**NBU**	**NBU**
**H6**	Small	In maternity	Mixed with older children	**NBU**	**PW**	**NBU**	**NA**
**H7**	Large	Designated	Mixed with older children	**NBU**	PW/NBU **	**NBU**	**NBU**
**H8**	Small	In maternity	Mixed with older children	**NBU**	**PW**	**REFER**	**PW**
**H9**	Large	Designated	Separate room	**NBU**	**PW**	**PW**	**PW**
**H10**	Medium	Designated	Mixed with older children	**NBU**	**NBU**	**NBU**	**NBU**
**H11**	Small	In maternity	Mixed with older children	**NBU**	**NBU**	**NBU**	**NBU**
**H12**	Large	Designated	Mixed with older children	**NBU**	**PW**	**NBU**	**NBU**

Inborn: *Neonates born within the admitting hospital;* Out-born: *Neonates not born within the admitting hospital;* Referred: *Neonates referred from other hospitals;* NBU **:** *Routinely admitted to the newborn unit;* PW: *Routinely admitted to the paediatric ward;* PW/NBU: *Routinely admitted to either paediatric ward or newborn unit;* REFER: *Not admitted, instead referred to other hospitals;* NA: *Not applicable* * All neonates who were more than 10 days old on the day of admission were admitted to PW unless they had jaundice; ** All neonates from the community who were more than 24 hours old at admission were admitted to PW apart from preterm neonates; ƚ Ward admission depended on the admitting clinician; § Referred neonates who were diagnosed with sepsis were admitted to the PW and those without sepsis were admitted to NBU

### Organisation of care

A relationship between the physical layout and infrastructure of the NBU and PW and the distribution of newborns between the two wards was observed. Hospitals (n=4) with a medium-large designated NBU ( Table 1) admitted fewer (12.5%) newborns to the PW compared to hospitals (n=8) where the NBU was small and part of the maternity ward or PW (22.0%). However, there was little consistency of reported admission policy based on physical layout of the PW and NBU ( Table 1).

## Discussion

Our study describes, for the first time, neonatal admissions to both NBUs and PWs across a network of public hospitals that would typically be the first referral level for women with complicated pregnancies or for sick or preterm newborns in Kenya. On average, 35.8% of all paediatric admissions were aged ≤28 days and 16.2% of these neonates were admitted to PWs. Previous reports suggest that neonates are making up an increasing proportion of paediatric admissions in Kenya, yet important quality gaps exist for this patient group ^11, 12^.

The practice of admitting neonatal patients to PWs is anecdotally described in healthcare settings internationally. However, the contribution of this patient group to overall neonatal and paediatric admissions is not well described in the literature. Where research has been conducted within resource-limited healthcare environments, concerns have been raised about the quality of care that neonates receive in this non-specialised paediatric setting where care may focus on the needs of older patient groups ^4, 5^. As part of efforts to address the persistently high mortality among newborns in low-income countries, it will be important to ensure that small and sick newborns are receiving care in the most appropriate setting with access to specialised staff and equipment ^1, 3^.

We observed inconsistencies in admission practices across hospitals related, at least in part, to the existing physical capacity and organisation of neonatal services. System-wide efforts to improve neonatal care could benefit from developing standardised policies, linking this to infrastructure and staff planning. It was observed that hospitals tended to admit outborn patients to PWs rather than NBUs. This is likely linked to infection prevention and control efforts. However, no formal policy on such an approach can be found for Kenya and some outborn newborns, specifically preterm newborns, are nonetheless often admitted to NBUs.

Our study finds that hospitals with larger designated NBUs were less likely to admit newborns on PW. One option to ensure newborns access specialised care, while also limiting infection, is to expand the capacity of NBUs to appropriately accommodate older and outborn newborns. However, NBUs in public sector hospitals in Kenya currently struggle with overcrowding and high patient to nurse ratios ^12^. Hence, such a policy would require strategic investment in space, including isolation rooms, and specific staff for different patient groups in many settings. Additional space and staff may also be required to accommodation kangaroo mother care services, which are now recommended for all newborns <2000g in Kenya. If consolidating neonatal care within NBU is not the preferred option then similar investments will be needed to make PWs more appropriate for neonatal care.

A limitation of our study is that information on admission policies was not collected during the same timeframe as admissions data. However, we do not expect that policies would have changed since the data collection period. Outcome data were missing for 31% of NBU admissions. Our estimates of mortality must, therefore, be interpreted cautiously. This may also signal wider problems with NBU admission data; missing data on admissions would result in us underestimating the total burden of neonatal admissions.

## Conclusion

Neonatal patients, a vulnerable patient group, represent 36% of all paediatric admissions. A substantial proportion are admitted to PWs, where there may be challenges of delivering quality care. Reducing neonatal morbidity and mortality is likely to benefit from a comprehensive long-term strategy spanning the organisation and resourcing of appropriate services that goes beyond local quality improvement efforts.

## Data availability

The source data are owned by the Kenyan Ministry of Health, County Governments and as the data might be used to de-identify hospitals the study authors are not permitted to share the source data directly. Users who wish to reuse the source data are able to make a request initially through the KEMRI-Wellcome Trust Research Programme data governance committee. This committee will supply contact information for the KEMRI Scientific and Ethical Review unit, County Governments and individual hospitals as appropriate. The KEMRI-Wellcome Trust Research Programme data governance committee can be contacted on: dgc@kemri-wellcome.org

## Supplementary material

Supplementary File 1: Calling script.
