# Supplementary material for: A hidden burden of neonatal illness? A cross-sectional study of all admissions aged less than one month across twelve Kenyan County hospitals
Source: Wellcome Open Res. 2018 Jan 30;2:119. Originally published 2017 Dec 18. [Version 2] doi: 10.12688/wellcomeopenres.13312.2 (PMC5771142; doi:10.12688/wellcomeopenres.13312.2)
Supplement: Supplementary file 1 [file wellcomeopenres-2-14990-s0000.tgz › e7de8a69-42dc-443a-9c92-31ad85021c04.docx]

**CALLING SCRIPT**

1. Introduce myself and say where I am calling from.
2. Explain briefly about my research
3. Seek consent to ask questions about the care of neonates in the hospital.
4. Proceed to ask questions

- Newborn care setting:
- Admission policies/guidelines of newborns in the hospital
- Admission of newborns to paediatric ward

1. Give them a chance to ask any questions/make clarifications
2. Thank them for the feedback

Hello, my name is Vivian Nyakangi calling from KEMRI Wellcome Trust Research Programme offices in Nairobi. I got your number from the Clinical Information Network (CIN) contacts.

Have you heard of CIN before?

(*If no, CIN is a collaborative network that was formed by the Ministry of Health in parterniship with Kemri Wellcome Trust Research Programme, Kenya Paediatric Association and 14 county (district) hospitals to support and improve the collection of Clinical Information that can be used to improve the quality of care for children. The information is collected by trained data clerks from Kemri Wellcome Trust Research Programme, who enter information daily from paediatric discharge records electronically. The information is then sent to Kemri offices for conducting researches and providing regular feedback to the hospitals).*

I am a post-graduate diploma student currently writing a thesis on neonates admitted to paediatric wards across CIN hospitals. I am therefore calling to better understand policies and practice in your hospital as regards neonatal admission.

Are you able to assist with the information?

*If yes,*

I will be asking a few questions,

1. What is the newborn care setting in your facility?

- Designated newborn unit
- Newborn unit in paediatric ward
- Newborn unit in maternity
- No newborn unit

1. Do you admit neonates to paediatric ward? *Yes/NO*

*If yes,*

1. What are some of the reasons why you would admit a newborn in paediatric ward and not in NBU?

*If no,*

1. What policies/guidelines do you follow when admitting/discharging neonates to the newborn care setting?
2. Where do you admit neonates brought from outside (home/other facilities) to your facility?
3. Do you admit all sick neonates born within your hospital in the newborn unit?
4. Do you have any questions or comments to make?

Thank you so much for taking your time to assist with the information.

*If No,*

Would it be better if I called back another time of can you refer me to someone else?

Thank you so much for your time.

**Data collection tool**

| **Name of hospital:** |
| --- |
| **Newborn care setting**: |
|  Designated newborn unit   Newborn unit in paediatric ward   Newborn unit in maternity   No newborn unit  Any other: |
| **Admission of neonates to paediatric ward?** |
| - **Yes** - **No** |
| **Reason for admitting babies to paediatric ward:** (*free text*) |
| **Policies and guidelines used for admission and discharge**: (*free text*) |
| **Extra comments** |
