# Supplementary material for: A hidden burden of neonatal illness? A cross-sectional study of all admissions aged less than one month across twelve Kenyan County hospitals
Source: Wellcome Open Res. 2018 Jan 30;2:119. Originally published 2017 Dec 18. [Version 2] doi: 10.12688/wellcomeopenres.13312.2 (PMC5771142; doi:10.12688/wellcomeopenres.13312.2)
Supplement: Peer review history

# Peer review: Referee response for version 1

**DOI:** 10.21956/wellcomeopenres.14445.r29113

**Published:** 2018-01-12

**Authors:** Fatima Gohar

**Affiliations:** 1 Eastern and Southern Africa Regional Office (ESARO), UNICEF, Nairobi, Kenya

This paper is clearly written, well-articulated and technically sound. As a reviewer, I found a mismatch between table 1 and narrative part (pg.5) “neonates who were referred from other hospitals .... without sepsis were admitted to NBU”, while table 1 shows that neonates who were referred from other hospitals were admitted to NBUs in 7/12 hospitals except of H2 and H 4. I would request to recheck this part.

I have read this submission. I believe that I have an appropriate level of expertise to confirm that it is of an acceptable scientific standard.

# Response

**Published:** 2018-01-18

**Authors:** Georgina Murphy

**Affiliations:** University of Oxford, UK

Dear Fatima Gohar,

Many thanks for your very helpful review. We very much appreciate your time and expertise.

Thank you for picking up on the error in numbers of hospitals admitting referred newborns to the NBU. Table 1 is correct and the text has been updated accordingly in version 2 to indicate 7/12 hospitals admitting referred newborns to the NBU. We have further clarified this by including the new text underlined below:

*Neonates who were referred from other hospitals were admitted to NBUs in 7/12 hospitals and to the PW in 2/12 hospitals. Admission of referred neonates depended on their age and condition in a further two hospitals. For example, in one hospital (H2), referred neonates who were diagnosed with sepsis were admitted to the PW and those without sepsis were admitted to NBU.*

Kind regards,

Georgina Murphy

# Peer review: Referee response for version 1

**DOI:** 10.21956/wellcomeopenres.14445.r29110

**Published:** 2018-01-09

**Authors:** Kathy Burgoine

**Affiliations:** 1 Neonatal Unit, Department of Paediatrics and Child Health, Mbale Regional Referral Hospital, Mbale, Uganda; 2 Mbale Clinical Research Institute, Mbale Regional Referral Hospital, Mbale, Uganda

Thank you for the opportunity to review this interesting and original paper from Kenya. It addresses the very real need to improve neonatal care in health facilities in Kenya and in similar low-resource settings (LRSs) if neonatal mortality is to be reduced. This paper highlights clearly that there need to improve availability and accessibility to specialist neonatal care in many hospitals in LRSs and that unfortunately many neonates are still cared for in non-specialist PWs. It also emphasises the need for consistency and guidance in admission policies of neonates in LRSs. This is a well written and highly relevant paper, which requires only minor revisions highlighted below.

 Please ensure consistency of terminology throughout, ideally using neonate/neonatal in preference to newborn. Again please ensure consistency when using NBU and PW in plural form. Preterm is normally defined as <37 weeks completed gestation not <36 weeks.

 It is interesting that the outcomes of 100% of neonatal patients are known for PW admissions and only for 69% of those admitted to NBUs. Given that these data came from the same county hospitals I wonder why the data collection was so much more complete in the PWs. I would like to see the possible reasons for this discussed in more detail. It is surprising that the mortality for the neonatal admissions to PWs was similar to the NBUs, one would expect that without specialist equipment and neonatal nursing that the mortality for those admitted to the PWs would be higher. This unexpected outcome should be highlighted and discussed. It is possible that the unknown outcomes for the NBUs hid a large proportion of the neonatal mortality. Anecdotally that is what we experience in Uganda. This should also be discussed within the manuscript.

 How was the size of the NBU defined? It would be interesting to know this and if possible to know the bed capacity, the actual bed occupancy and the physical size of the NBUs. If possible it would useful to provide the broad criteria for admission of neonates to these county hospitals whether it was to the NBU/PW. That is did they have a weight or gestational cut-off for admissions, did they admit all neonates with jaundice or only requiring phototherapy, all cases of sepsis or only severe etc? This information would allow readers to better compare to their situation to this paper.

 It would be good to see some discussion on the evidence for admitting outborn neonates to the PWs and not to the NBU. 

I have read this submission. I believe that I have an appropriate level of expertise to confirm that it is of an acceptable scientific standard.

# Response

**Published:** 2018-01-18

**Authors:** Georgina Murphy

**Affiliations:** University of Oxford, UK

Dear Kathy Burgoine,

Thank you very much for your helpful and insightful feedback on our paper. We very much appreciate your time and expertise. We have provided individual responses to each of your comments (your comments are in bold) below and have made changes to the paper for version 2 (underlined text).

**Please ensure consistency of terminology throughout, ideally using neonate/neonatal in preference to newborn. Again please ensure consistency when using NBU and PW in plural form. Preterm is normally defined as <37 weeks completed gestation not <36 weeks.**

Thank you, we have amended these throughout.

**It is interesting that the outcomes of 100% of neonatal patients are known for PW admissions and only for 69% of those admitted to NBUs. Given that these data came from the same county hospitals I wonder why the data collection was so much more complete in the PWs. I would like to see the possible reasons for this discussed in more detail.**

The sources of data for the PW and the NBU were different, which led to differences in quality of the data we were able to obtain. The PW data were extracted from the medical records in hospitals that are part of the Clinical Information Network, a network that aims to improve documentation. By contrast, the NBU data were extracted from the admission register, for which no intervention to improve documentation has been made. In addition, we have found in previous work, that data recorded in admission registers for newborns tends to be considerably more sparse that that found in medical records/clinical notes. We have mentioned this in the limitations and also included the following text in the discussion:

*We report similar neonatal mortality for NBUs and PWs (12.3% and 11.1%, respectively) in this study. These rates should, however, be interpreted with caution given the large degree of missing data from NBUs. It is possible that a considerable amount of neonatal mortality is missing due to failure to record outcomes in NBU admission registers for almost a third of patients.*

**It is surprising that the mortality for the neonatal admissions to PWs was similar to the NBUs, one would expect that without specialist equipment and neonatal nursing that the mortality for those admitted to the PWs would be higher. This unexpected outcome should be highlighted and discussed. It is possible that the unknown outcomes for the NBUs hid a large proportion of the neonatal mortality. Anecdotally that is what we experience in Uganda. This should also be discussed within the manuscript.**

Indeed, given the large amount of missing data on mortality in the NBU, these data should be interpreted with caution. As you have rightly pointed out, it is likely that the missing data are hiding a large proportion of mortality. However, there are a few factors that may contribute to differences or similarities in mortality. It is true that the less specialised environment of the PW would lead us to expect poorer specialised care and hence higher mortality. On the other hand, however, the newborns admitted to the NBU tend to be younger than those admitted to the PW and most hospitals admitted preterm newborns to the NBU, even if ‘outborn’. Given that neonatal mortality decreases with age and conditions due to preterm birth are major contributors to neonatal death in this setting, we may expect mortality in the NBU to be higher than in the PW. These two conflicting effects may work to cancel one another out, leading to recorded similar neonatal mortality on the two wards. It is certainly an interesting point worth highlighting, so we have included the following in the discussion section:

*It is expected that neonates receiving less specialised care on PWs may have worse outcomes than those admitted to specialised NBUs. Our study has shown, however, that a simple comparison of mortality on PWs and NBUs is not a fair one given the difference in admission policies for neonates to these two wards. In particular, neonates admitted to NBUs tend to be considerably younger and more likely to be preterm births; hence, increasing their risk of mortality by comparison to their older term-born counterparts in PWs.*

**How was the size of the NBU defined? It would be interesting to know this and if possible to know the bed capacity, the actual bed occupancy and the physical size of the NBUs.**

The size of NBUs was defined in terms of the physical space rather than admission or bed numbers. These sizes are estimated based on experience of visiting the NBUs. Unfortunately, we do not have data on bed capacity, occupancy or measured physical size. However, if you are interested, in a recent study (Nairobi Newborn Study) we did obtain detailed information on bed capacity and occupancy for all NBUs in Nairobi City County. The work is under peer review at PLOS One. I am happy to share details. Furthermore, information on the facilities in Kenya is available from the Master Facility List ( http://kmhfl.health.go.ke), however, the number of cots recorded appear to be out of date and are not disaggregated by ward.

**If possible it would useful to provide the broad criteria for admission of neonates to these county hospitals whether it was to the NBU/PW. That is did they have a weight or gestational cut-off for admissions, did they admit all neonates with jaundice or only requiring phototherapy, all cases of sepsis or only severe etc? This information would allow readers to better compare to their situation to this paper.**

We agree that such information would be very helpful. However, such detailed policies currently don’t exist for hospitals in Kenya. Admission decisions tend, instead, to be up to individual clinicians and general hospital norms. An exception is the new national policy on admitting all <2000 g newborns for kangaroo mother care, as mentioned in the discussion section (though to-date there has been little active implementation of this policy). In recognition of this limitation and as part of the above mentioned study (Nairobi Newborn Study), we held stakeholder meetings to capture agreement on what newborns should and are routinely being admitted to hospitals in Kenya. The outcome of this work is published here: http://gh.bmj.com/content/2/4/e000472. We have included a brief explanation of this in the discussion:

*Despite a recent effort to gain consensus among stakeholders on major neonatal conditions for admission to Kenyan hospitals, ^13 ^there is currently an absence of written policy in many facilities on when and where to admit, refer, or discharge neonates.*

**It would be good to see some discussion on the evidence for admitting outborn neonates to the PWs and not to the NBU.**

Unfortunately, we have been unable to find any literature on this very important issue, apart from two papers (Simiyu et al and Okposio et al) that report the mortality and morbidity of newborns admitted to general PWs. We welcome any suggestions from others who may be familiar with other work on this topic. We have further highlighted the findings of these two studies in the discussion:

*Previous studies of neonatal admissions to PWs in sub-Saharan Africa have found mortality rates as high as 20.3% and 31.5%. ^4,5^*
